# Oral Probiotics in *Acne vulgaris*: A Systematic Review and Meta-Analysis of Double-Blind Randomized Clinical Trials

**DOI:** 10.3390/medicina61122152

**Published:** 2025-12-03

**Authors:** Jeng-Wei Tjiu, Chia-Fang Lu

**Affiliations:** 1 Department of Dermatology, College of Medicine, National Taiwan University Hospital, National Taiwan University, Taipei 100, Taiwan; 2 Grace Smile Dental Clinic, Taipei 106, Taiwan

**Keywords:** *Acne vulgaris*, oral probiotics, *Lactobacillus*, *Bifidobacterium*, gut–skin axis, microbiome, randomized controlled trials, systematic review, meta-analysis

## Abstract

*Background and Objectives: Acne vulgaris* is a prevalent chronic inflammatory skin condition affecting adolescents and young adults worldwide. Increasing concern regarding antimicrobial resistance has renewed interest in microbiome-modulating therapies, including oral probiotics. This systematic review and meta-analysis evaluated the efficacy and safety of oral probiotic supplementation for *acne vulgaris* using contemporary random-effects methods. *Materials and Methods:* Following PRISMA 2020 guidelines, we searched PubMed, Embase, Web of Science, and ClinicalTrials.gov through November 2025 without language restrictions. Eligible studies were double-blind randomized controlled trials (RCTs) comparing oral probiotics with placebo or standard therapy for ≥4 weeks and reporting quantitative acne severity outcomes. Risk of bias was assessed using RoB 2.0. Standardized mean differences (SMDs) were pooled using restricted maximum likelihood (REML) with Hartung–Knapp adjustment. Heterogeneity was summarized using I^2^, τ^2^ (95% CI), and 95% prediction intervals. Adverse events were extracted. *Results:* Three RCTs (*n* = 231) met eligibility criteria. Pooled analysis suggested a modest reduction in inflammatory lesion counts favoring probiotics (SMD −0.57; 95% CI −0.94 to −0.21), although heterogeneity was substantial (I^2^ = 72%; τ^2^ = 0.11). The 95% prediction interval (−1.25 to 0.11) indicated that future studies may plausibly observe no meaningful effect. Sensitivity analyses using the DerSimonian–Laird estimator produced comparable results. All trials reported good short-term tolerability with no serious adverse events. Risk of bias was low in two trials and of some concern in one. Certainty of evidence was rated low to moderate. *Conclusions:* Oral probiotics may modestly reduce acne severity as a generally safe, antibiotic-sparing adjunct; however, the current evidence base is small and heterogeneous, and the certainty of effect remains low-to-moderate. Larger, standardized RCTs are required before firm clinical recommendations can be made. Registration: PROSPERO CRD420251181388. Funding: This research received no external funding.

## 1. Introduction

*Acne vulgaris* is among the most prevalent chronic inflammatory dermatologic conditions worldwide and remains a leading reason for dermatology consultations among adolescents and young adults. In addition to visible manifestations such as comedones and inflammatory papules, and pustules, acne imposes a substantial psychosocial burden, adversely affecting self-esteem, social functioning, and overall quality of life [1]. Traditional pathogenic models highlight follicular hyperkeratinization, excess sebum production, proliferation of *Cutibacterium acnes*, and subsequent inflammation of the pilosebaceous unit [2]. More recently, a broader systemic framework has emerged, linking cutaneous inflammation to host metabolic and microbial homeostasis—particularly through the gut–skin axis [3].

### 1.1. Clinical Significance of Acne

Acne is one of the most common dermatologic diseases globally and represents a major public health concern because of its high frequency, chronic and relapsing course, and substantial psychosocial impact. Epidemiologic studies indicate that up to 85–95% of adolescents and 40–55% of adults experience acne, making it one of the most frequently encountered conditions in both primary care and dermatology practice [4,5,6]. The American Academy of Dermatology and international guideline panels recognize acne as a disorder with major clinical, psychological, and economic consequences, comparable to other chronic inflammatory diseases such as asthma and arthritis [7].

Clinically, acne can lead to permanent sequelae, including atrophic and hypertrophic scarring and dyspigmentation, which may persist long after active inflammation has subsided [7]. Scarring risk is strongly associated with delayed or suboptimal treatment, severe inflammatory phenotypes, and repeated cycles of untreated lesions [7]. Acne may also result in persistent erythema and post-inflammatory hyperpigmentation—particularly in individuals with skin of color—and long-lasting alterations in skin texture, all of which can further compromise quality of life [7].

Beyond physical manifestations, acne has well-documented psychosocial effects. Numerous studies demonstrate associations between acne and depression, anxiety, social withdrawal, and reduced self-esteem [8]. Severe acne and acne scarring have been linked to higher rates of suicidal ideation, emphasizing the importance of timely and effective management [9]. The psychosocial burden can be disproportionate to objective clinical severity, especially in young adults and in women with adult-onset acne [7,9,10]. Economically, acne contributes to significant healthcare utilization—including physician visits, prescription medications (e.g., topical retinoids, antibiotics, hormonal therapies, isotretinoin), over-the-counter products, and procedural interventions—with global direct and indirect costs estimated to exceed several billion US dollars annually [7,10].

From a broader clinical standpoint, acne may also serve as a marker of underlying systemic or hormonal abnormalities, such as hyperandrogenism, polycystic ovary syndrome, medication-induced eruptions (e.g., from epidermal growth factor receptor inhibitors or systemic corticosteroids), and environmental or occupational exposures such as dioxins or polychlorinated biphenyls (chloracne) [1,2,7]. Recognizing these associations is essential because acne can sometimes be the earliest or most visible sign of systemic disease.

Taken together, acne is not only highly prevalent but also a condition with enduring physical, psychological, and economic consequences. Early recognition, accurate assessment of severity, and evidence-based treatment are crucial to minimize long-term sequelae and improve patient well-being.

### 1.2. Recent Updates in Treatment Strategies

Contemporary acne management continues to evolve, with several major updates reflected in the 2024 American Academy of Dermatology (AAD) Clinical Practice Guideline and recent editions of leading dermatology textbooks [7,11]. These evidence-graded recommendations (GRADE methodology) emphasize antimicrobial stewardship, multimodal therapy, and individualized patient-centered management [7].

Topical therapies remain the foundation of treatment, with strong recommendations for retinoids (adapalene, tretinoin, tazarotene, and the RAR-γ–selective trifarotene), benzoyl peroxide (BPO), and fixed-dose combinations of BPO with either a retinoid or a topical antibiotic. To prevent antimicrobial resistance, topical antibiotics are recommended only in combination with BPO and should not be used as monotherapy. Newly approved agents such as clascoterone 1% cream, an androgen receptor inhibitor, provide additional options for inflammatory acne and have been incorporated into guideline-based first-line algorithms [7].

Systemic antibiotics, including doxycycline, minocycline, and sarecycline, are recommended for moderate-to-severe inflammatory acne, but with strict limitations on duration. Current guidelines emphasize time-limited courses, always combined with topical BPO and/or a retinoid, and discourage the use of oral erythromycin (except during pregnancy) as well as all forms of antibiotic monotherapy [7].

Hormonal therapies play a central role in female patients. Combined oral contraceptives and spironolactone (50–200 mg/day) have strong evidence supporting their use. Notably, the 2024 guideline highlights that spironolactone demonstrates comparable clinical effectiveness to oral tetracyclines for many women and clarifies that routine potassium monitoring is not required except in select high-risk populations [7].

Oral isotretinoin remains strongly recommended for severe nodular acne, scarring acne, and cases refractory to standard therapy. Updated guidance favors more flexible laboratory monitoring and acknowledges variation in cumulative dosing, while retaining a target of approximately 120–150 mg/kg for long-term disease control [7].

Finally, several adjunctive and emerging therapies are addressed. Conditional recommendations support the selective use of light-based therapies, laser modalities, photodynamic therapy, chemical peels, and dietary modification (particularly low glycemic load diets). Conversely, the guideline notes insufficient evidence to recommend most nutraceuticals and supplements—such as zinc, nicotinamide, or probiotics—as primary therapeutic agents, despite their widespread use [7].

Together, these updates reflect an increased emphasis on antimicrobial stewardship, evidence-based topical combinations, judicious use of systemic agents, and personalized multimodal care [7].

### 1.3. Prevalence of Acne vulgaris Compared with Other Acne Subtypes

*Acne vulgaris* is by far the most common acne subtype, accounting for well over 90–95% of all acne presentations across age groups [4,5,6]. By contrast, other acne subtypes occur relatively infrequently and are often confined to specific risk groups or clinical contexts.

*Acne mechanica*, driven by chronic friction, pressure, and occlusion (e.g., from sports equipment, helmets, or tight masks), is relatively common in certain populations but still affects only a small proportion of the general population—estimated at 2–5%, with higher rates (up to 15–35%) among athletes, military personnel, or individuals with sustained mechanical irritation [12,13]. *Acne cosmetica*, historically reported in 3–5% of cosmetic users, has become less frequent (<2%) in settings where non-comedogenic formulations are widely used; it predominantly affects women and is characterized by comedonal eruptions on cosmetically treated areas [11,12].

Severe nodulocystic forms such as *acne conglobata* are rare, affecting <1% of acne patients, and are marked by deep nodules, draining sinus tracts, and a high risk of disfiguring scarring, particularly among young men and individuals exposed to anabolic steroids or other triggers [1,7,11,12]. *Acne fulminans* is an extremely rare, abrupt-onset, ulcerative variant—occurring in a small subset of adolescent males—and may be associated with systemic symptoms or isotretinoin exposure [1,7,11,12]. *Chloracne* is likewise rare and typically restricted to individuals with substantial exposure to halogenated aromatic hydrocarbons (e.g., dioxins, polychlorinated biphenyls), where it serves as a cutaneous marker of toxic exposure [14]. *Drug-induced acneiform eruptions*, including those associated with corticosteroids, EGFR inhibitors, and certain antiepileptics, are characterized by monomorphic inflammatory papules and pustules without comedones and occur in a minority of treated patients, although incidence can be high (up to 60–80%) in specific contexts such as EGFR inhibitor therapy [15,16].

In summary, *acne vulgaris* overwhelmingly dominates the clinical landscape, whereas other acne subtypes—*acne mechanica*, *acne cosmetica*, *acne conglobata*, *acne fulminans*, *chloracne, and drug-induced acneiform eruptions*—each account for only a small fraction of cases and are often linked to particular exposures or systemic conditions. This distribution justifies a primary focus on *acne vulgaris* when evaluating therapeutic strategies and prognostic factors in population-based and interventional studies [12].

### 1.4. Rationale for Microbiome-Directed Therapies

Growing evidence suggests that intestinal dysbiosis may amplify systemic inflammatory signaling via immune and neuroendocrine pathways, including circulating endotoxins and cytokine cascades [3,17,18]. In patients with acne, prolonged use of systemic antibiotics can disrupt gut microbial composition and promote alterations in the cutaneous microbiome, potentially perpetuating inflammatory responses rather than achieving durable remission [19,20]. Restoration of microbial balance through oral probiotics has therefore attracted interest as a potential antibiotic-sparing strategy that may modulate systemic and cutaneous inflammation while preserving commensal microbial diversity [21,22,23,24].

### 1.5. Limitations of Conventional Therapy

Oral antibiotics—most commonly tetracyclines and macrolides—remain mainstays in the management of moderate-to-severe acne but are associated with important limitations, including gastrointestinal adverse effects, photosensitivity, and the promotion of antimicrobial resistance in both *C. acnes* and off-target commensal flora [7,19,20,21]. Contemporary acne guidelines, including those from the Global Alliance to Improve Outcomes in Acne and the 2024 American Academy of Dermatology (AAD) guideline, strongly recommend restricting antibiotic courses to the shortest effective duration (typically ≤12 weeks) and combining systemic antibiotics with topical benzoyl peroxide and/or retinoids to mitigate resistance [1,7,21]. Despite these recommendations, real-world data indicate that prolonged and repeated antibiotic courses remain common [1,7,21]. In this context, adjunctive therapies that can reduce inflammatory lesions and improve clinical outcomes without contributing to antimicrobial resistance are of high clinical relevance [1,7,21].

### 1.6. Evidence Landscape for Probiotics in Acne

Over the past decade, several small randomized and non-randomized clinical studies have evaluated the potential effects of oral probiotics—most commonly involving *Lactobacillus* and *Bifidobacterium* strains—on acne severity, sebum production, systemic inflammatory mediators, and skin barrier parameters [25,26,27,28]. Collectively, these investigations, supported by mechanistic work, suggest that probiotics may influence acne-related pathways through modulation of local and systemic immune responses, enhancement of epithelial barrier integrity, and alterations in gut and skin microbiota composition [25,26,27,28,29,30]. However, these proposed mechanisms remain incompletely understood, and the certainty of evidence is limited.

The existing clinical evidence base is constrained by small sample sizes, heterogeneous study designs, and substantial variability in probiotic formulations, including differences in strain identity, colony-forming unit (CFU) counts, delivery matrices, and treatment durations. Outcome measures have also been inconsistent, with non-standardized lesion-based counts or global severity scores limiting comparability across studies.

Previous meta-analyses have attempted to synthesize these findings but have been restricted by the paucity and heterogeneity of available trials and have relied largely on the conventional DerSimonian–Laird random-effects estimator [31]. This method is known to underestimate between-study variance when the number of included studies is small, which may produce overly narrow confidence intervals and spuriously precise estimates [32]. These limitations underscore the need for an updated quantitative synthesis using more robust variance-estimation approaches and for a clearer accounting of heterogeneity and uncertainty.

In this context, contemporary random-effects methods such as restricted maximum likelihood (REML) with Hartung–Knapp adjustment provide improved variance estimation and interval calibration for sparse datasets [33,34]. These methodological advances motivate the present review, which aims to reassess the evidence using modern synthesis techniques and to more explicitly evaluate heterogeneity, certainty, and clinical relevance [33,34].

### 1.7. Methodological Advances and Study Objective

Modern random-effects meta-analytic approaches, such as those based on restricted maximum likelihood (REML) estimation coupled with Hartung–Knapp adjustments, provide more reliable variance estimation and improved coverage of confidence and prediction intervals, particularly in the setting of sparse or heterogeneous evidence [33,34,35]. Consistent with recommendations from the Cochrane Handbook and recent methodological guidance [36], we conducted an updated systematic review and meta-analysis focusing exclusively on double-blind randomized controlled trials of oral probiotics for acne vulgaris.

The primary objectives of this study were to (i) estimate the pooled effect of oral probiotic supplementation on clinically assessed acne severity, (ii) quantify between-study heterogeneity and explore potential sources of variability, and (iii) appraise the overall certainty and real-world applicability of the current evidence base. By applying contemporary synthesis methods and explicitly addressing issues of heterogeneity, risk of bias, and regulatory considerations, we aim to provide a balanced and clinically meaningful assessment of the role of oral probiotics as adjunctive therapy in acne management.

## 2. Materials and Methods

### 2.1. Protocol and PRISMA Compliance

**Figure 1:**
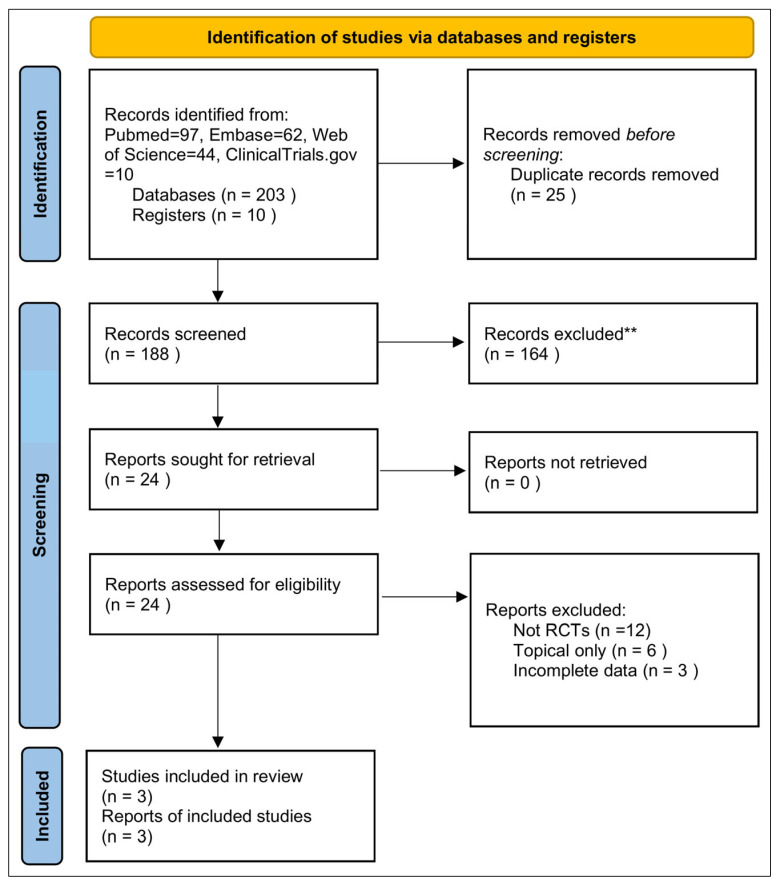
PRISMA 2020 flow diagram summarizing the study selection process for the systematic review [37]. ** Records excluded at the screening stage (*n* = 164) were excluded because they were non-RCTs, topical-only probiotic studies, review articles, mechanistic studies, or lacked extractable quantitative acne outcomes.

This systematic review and meta-analysis was conducted in accordance with the Preferred Reporting Items for Systematic Reviews and Meta-Analyses (PRISMA) 2020 statement [37]. The review protocol was prospectively registered in PROSPERO (Registration ID: CRD420251181388). The PRISMA 2020 flow diagram summarizing the identification, screening, eligibility, and inclusion of studies is presented as Figure 1. A completed PRISMA 2020 Checklist (Appendix A) and PRISMA Abstract Checklist (Appendix B) are included [37].

### 2.2. Eligibility Criteria

Studies were selected according to pre-specified inclusion and exclusion criteria structured around the PICOS framework: Population: Humans diagnosed with *acne vulgaris* (any severity), confirmed by clinical criteria. Intervention: Oral probiotics, any strain(s), formulation, or dose. Comparator: Placebo or standard acne therapy (e.g., topical treatments). Outcomes: Quantitative acne severity outcomes (e.g., inflammatory lesion count, non-inflammatory lesions, total lesion count, global acne scales). Study Design: Double-blind randomized controlled trials (RCTs) lasting ≥4 weeks. Exclusion criteria: Non-randomized studies, open-label or single-blind trials, topical probiotics, synbiotic/prebiotic-only studies, animal or in vitro studies, lack of extractable numeric outcomes, and non–non-peer-reviewed abstracts.

### 2.3. Information Sources and Search Strategy

A comprehensive systematic search was conducted in PubMed, Embase, Web of Science, and ClinicalTrials.gov from database inception through November 2025. No language or geographic restrictions were applied. The search strategy combined controlled vocabulary (MeSH/Emtree) and free-text terms related to acne and probiotics, including: “*acne vulgaris*”, “*acne*”, “*probiotic*” *, “*Lactobacillus*”, “*Bifidobacterium*”, “*gut–skin axis*”, “*microbiome*”, “*randomized*”, “*double-blind*”. Full database-specific search strings are provided in Appendix C [37].

Backward and forward citation tracking of eligible articles was performed. Trial registries were examined for unpublished or ongoing studies.

### 2.4. Study Selection Process

All identified records were imported into EndNote X9 for duplicate removal and then screened using Rayyan. Two independent reviewers screened the titles and abstracts. Full-text articles were subsequently reviewed for eligibility by the same reviewers. Disagreements were resolved through discussion between reviewers. The full selection process is depicted in the PRISMA 2020 flow diagram (Figure 1) [37].

### 2.5. Data Extraction

A standardized data extraction form was used to collect study characteristics (year, country, sample size); participant demographics; *Acne* severity and diagnostic criteria; probiotic strain(s), dose, formulation, and duration; comparator details; outcome measures and time points; adverse events; funding source and conflicts of interest. Two reviewers independently extracted data, and discrepancies were resolved by consensus. When necessary, study authors were contacted for clarification or missing data.

### 2.6. Risk of Bias Assessment

Risk of bias for each RCT was assessed using the Cochrane Risk of Bias 2.0 (RoB 2.0) tool, evaluating: Randomization process; Deviations from intended interventions; Missing outcome data; Outcome measurement; Selection of reported results. Assessments were performed independently by two reviewers. Results are summarized in Table 1 [38].

### 2.7. Effect Measures

For continuous outcomes, standardized mean differences (SMD) using Hedges g were calculated. Corresponding 95% confidence intervals (CI) were extracted or derived from reported summary statistics. When not directly provided, means and standard deviations were reconstructed from medians, ranges, or interquartile ranges using validated methods (standard meta-analytic conversions; see Cochrane Handbook) [36].

### 2.8. Data Synthesis and Statistical Analysis

The primary quantitative synthesis used a random-effects model based on Restricted Maximum Likelihood (REML) with Hartung–Knapp (HK) adjustment for more accurate variance estimation under small sample sizes [18,19].

Secondary sensitivity analyses included: DerSimonian–Laird (DL) random-effects estimator [33,34]; Leave-one-out (LOO) influence analysis; Alternative variance estimators (Paule–Mandel) [32,35]; Cumulative meta-analysis by publication year; Statistical heterogeneity was assessed using: I^2^ (percentage of variation due to heterogeneity) [39]; τ^2^ (between-study variance, with 95% CI using Q-profile) [32,35]; Cochran’s Q test; 95% Prediction Interval (PI) to estimate expected variation in future studies [36]. Publication bias was evaluated using funnel plot visualization and Egger’s regression test (*p* < 0.05 suggestive) [40]. All analyses were conducted using R (metafor package).

### 2.9. Reporting Bias Assessment

Risk of publication bias and small-study effects was evaluated using funnel plots, regression-based asymmetry testing (Egger’s test), and comparison of REML-HK vs. DL estimators [40,41].

### 2.10. Certainty of Evidence

The certainty of evidence was evaluated using the GRADE framework, considering: Risk of bias; Inconsistency; Indirectness; Imprecision; Publication bias [42]. Final ratings (high, moderate, low, or very low certainty) are provided in Table 2 [42].

## 3. Results

### 3.1. Study Selection

The database search identified a total of 213 records (203 from databases and 10 from registers). After removal of 25 duplicate records, 188 records remained for title and abstract screening. Of these, 164 records were excluded.

A total of 24 reports were sought for retrieval, and all 24 were successfully retrieved. These reports were assessed for eligibility according to the predefined inclusion and exclusion criteria.

All excluded full-text reports are listed in Supplementary Table S1 [3,18,26,27,28,29,43,44,45,46,47,48,49,50,51,52,53,54,55,56,57]. Reasons for exclusion were: Not RCTs (n = 12), Topical only (n = 6), and Incomplete data (n = 3).

Ultimately, three double-blind randomized controlled trials (n = 231 participants) met all inclusion criteria and were included in the qualitative and quantitative synthesis. A PRISMA 2020 flow diagram summarizing the identification, screening, eligibility assessment, and inclusion process is shown in Figure 1.

### 3.2. Study Characteristics

The three included double-blind randomized controlled trials were published between 2021 and 2025 and were conducted in Asia and Europe. Sample sizes ranged from 61 to 90 participants, and intervention durations varied between 8 and 12 weeks.

The probiotic formulations differed substantially across studies. Interventions consisted of either single-strain products (e.g., *Lactobacillus paracasei*) or multi-strain combinations (e.g., *Lactobacillus plantarum*, *Bifidobacterium lactis*, *Lactobacillus acidophilus*), with notable variability in strain composition, viability, dosing, and delivery matrices. Comparator groups received either placebo capsules or concomitant topical therapy, factors that may influence generalizability and contribute to clinical heterogeneity.

All trials assessed inflammatory lesion counts as the primary outcome. Secondary outcomes included non-inflammatory lesions, total lesion burden, validated global acne severity scales, and safety/tolerability endpoints.

Detailed characteristics of each individual trial—including study location, sample size, probiotic formulation, dosing regimen, and outcome assessment timing—are summarized in Table 3.

### 3.3. Risk of Bias Assessment

Risk-of-bias evaluations using RoB 2.0 showed: Two studies (Kim 2021; Eguren 2024) were rated low risk of bias across all domains. One study (Atefi 2025) demonstrated some concerns, mainly due to minor deviations from intended interventions and outcome measurement considerations [22,23,24,38]. A full domain-level summary is provided in Table 1 [38].

### 3.4. Effects on Acne Severity (Primary Outcome)

The change in inflammatory lesion counts was synthesized using a random-effects model based on restricted maximum likelihood (REML) with Hartung–Knapp adjustment. The pooled standardized mean difference indicated a modest reduction in inflammatory lesions favoring oral probiotics (SMD −0.57; 95% CI −0.94 to −0.21).

**Figure 2:**
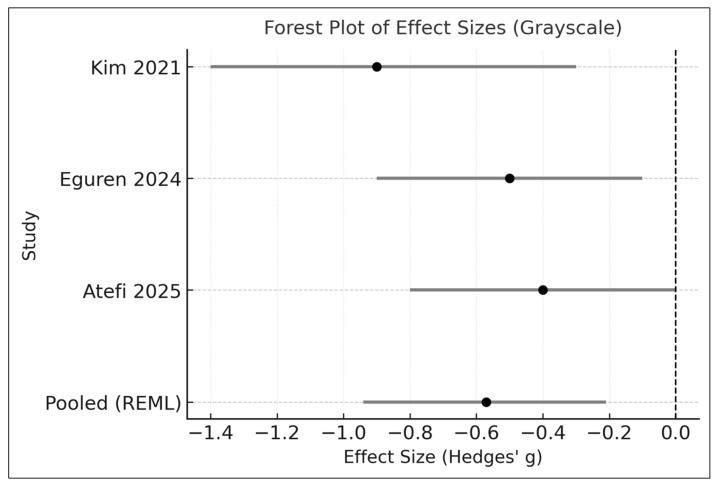
Forest plot presenting the pooled standardized mean difference (SMD) in inflammatory lesion counts comparing oral probiotic supplementation with control across the three included double-blind randomized controlled trials. Effect sizes were synthesized using a random-effects model with restricted maximum likelihood (REML) estimation and Hartung–Knapp adjustment. Horizontal lines represent 95% confidence intervals.

However, between-study heterogeneity was substantial (I^2^ = 72%; τ^2^ = 0.11), and the 95% prediction interval (−1.25 to 0.11) suggested that the true effect in future settings may plausibly range from a large reduction to no meaningful benefit. These findings therefore warrant cautious interpretation, particularly given the small number of available trials and the pronounced clinical and methodological variability across studies—including differences in strain composition, dosing regimens, baseline acne severity, and the use of adjunctive therapies. The forest plot summarizing individual and pooled effect sizes is shown in Figure 2.

### 3.5. Sensitivity and Additional Analyses

#### 3.5.1. DerSimonian–Laird Comparison

Applying the conventional DerSimonian–Laird estimator yielded a similar pooled effect (SMD −0.58), although this method is known to underestimate between-study variance when the number of trials is small. The agreement in direction and magnitude provides supportive—but not definitive—evidence of estimator robustness.

#### 3.5.2. Leave-One-Out (LOO) Influence Analysis

Leave-one-out analyses produced pooled SMD values ranging from −0.53 to −0.62. Although removal of any single trial did not reverse the direction of the effect, the magnitude of change underscores the influence of individual study characteristics, given the limited evidence base (k = 3). These findings further emphasize the need for cautious interpretation.

#### 3.5.3. Cumulative Meta-Analysis

Cumulative synthesis (2021 → 2024 → 2025) demonstrated progressively narrower confidence intervals and a stable direction of effect. However, because this pattern is driven by only three available trials, the apparent “convergence” should not be interpreted as definitive confirmation of efficacy.

#### 3.5.4. Subgroup Exploration (Descriptive Only)

Given the limited number of trials, subgroup analyses are descriptive and hypothesis-generating only.

(1) Multi-strain vs. single-strain formulations

Multi-strain combinations demonstrated numerically larger reductions in inflammatory lesions compared with the single-strain formulation. This pattern is biologically plausible but cannot be confirmed statistically.

(2) Monotherapy vs. adjunctive therapy

Effect directions were similar across monotherapy and adjunctive settings, although differences in baseline severity and concomitant treatments limit interpretability.

(3) Geographic region

Trials from Asia and Europe showed broadly similar effect directions; however, geographic inference is constrained by the sparse evidence base.

(4) Interpretation Summary

Collectively, these exploratory comparisons highlight several potential modifiers of probiotic response—including strain composition, delivery matrices, baseline severity, and use of co-interventions—but are insufficient to support firm conclusions. These descriptive patterns likely reflect underlying clinical and methodological heterogeneity that could not be formally evaluated with only three trials.

Taken together, these design differences provide a plausible explanation for the substantial statistical heterogeneity (I^2^ = 72%). Variation in probiotic strain composition (single- vs. multi-strain), dosing regimens, treatment duration, and concomitant topical or systemic therapies likely modulates both the magnitude and timing of clinical response. With only three trials, these patterns can only be described qualitatively; nonetheless, they underscore the need for future studies that explicitly compare standardized probiotic formulations across harmonized acne severity outcomes.

### 3.6. Publication Bias Assessment

**Figure 3:**
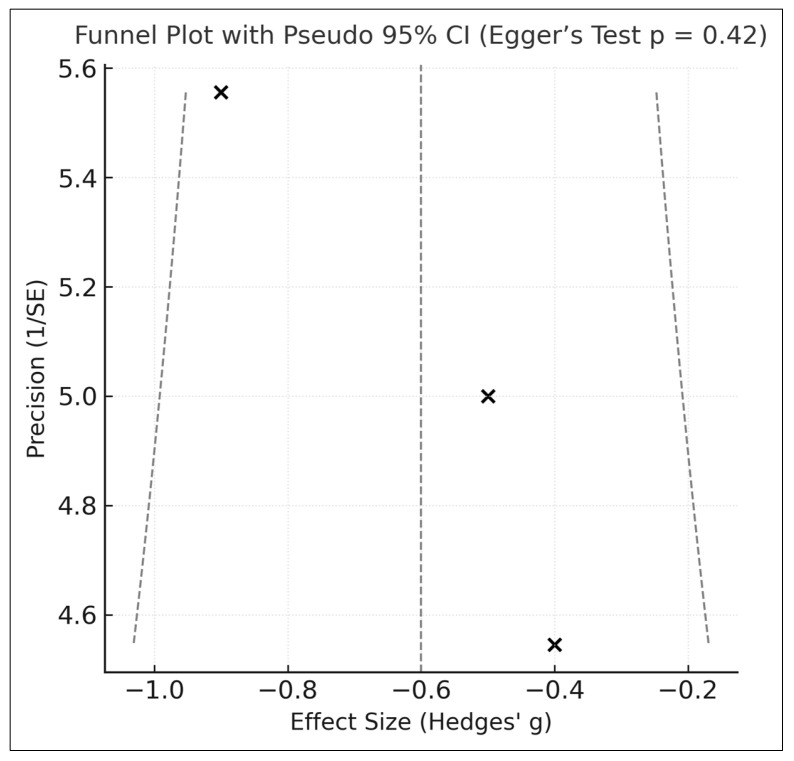
Funnel Plot With Pseudo 95% Confidence Intervals (Egger’s Test) [25,26].

Visual inspection of the funnel plot (Figure 3) did not reveal clear evidence of asymmetry. Egger’s regression test (*p* = 0.42) also showed no statistically significant indication of small-study effects; however, this test is substantially underpowered with only three included trials.

Accordingly, publication bias cannot be excluded, and both the apparent symmetry of the funnel plot and the non-significant Egger result should be interpreted with caution.

### 3.7. Adverse Events

Across all included trials, oral probiotics were generally well tolerated. Mild gastrointestinal symptoms—such as bloating or transient abdominal discomfort—were reported at comparable frequencies in probiotic and control groups. No serious adverse events were identified in any study.

However, given the small sample sizes and relatively short treatment durations of the included trials, definitive conclusions regarding rare, delayed, or long-term adverse effects cannot be drawn.

### 3.8. Certainty of Evidence

The overall certainty of evidence, assessed using the GRADE framework, was rated low-to-moderate. Downgrades were applied for inconsistency (I^2^ = 72%), imprecision (wide prediction interval and small total sample size), and potential small-study effects given the limited number of trials (k = 3).

No downgrading was required for risk of bias or indirectness, as the included RCTs were generally well designed and directly addressed the research question.

The complete GRADE evidence profile is presented in Table 2.

### 3.9. Safety and Tolerability

All included trials systematically monitored adverse events, and no serious events were identified. Mild gastrointestinal symptoms—occurring in fewer than 5% of participants—were reported at similar frequencies in probiotic and control groups.

In one adjunctive-therapy trial, probiotics appeared to lessen doxycycline-associated dyspepsia; however, given the small sample size and limited duration of follow-up, this finding should be interpreted as exploratory.

Overall, the short-term safety profile of oral probiotics was favorable, although the available data are insufficient to assess infrequent or long-term adverse effects.

### 3.10. Summary of Findings

Across the three double-blind randomized controlled trials (*n* = 231), oral probiotics were associated with a modest reduction in inflammatory acne severity relative to control. No major safety concerns were identified, and reported adverse events were mild and comparable between groups.

However, the evidence base is small and heterogeneous, with notable variability in probiotic strains, formulations, dosing regimens, co-interventions, and baseline patient characteristics. The 95% prediction interval (−1.25 to 0.11) encompassed the possibility of little to no effect, indicating that true responses may vary substantially across populations and settings.

Accordingly, these findings should be interpreted as preliminary and hypothesis-generating rather than definitive. Larger, standardized, and adequately powered randomized trials are needed to establish the magnitude, consistency, and clinical relevance of probiotic-associated benefits in acne management.

## 4. Discussion

This systematic review and updated meta-analysis synthesized evidence from three double-blind randomized controlled trials evaluating oral probiotic supplementation for acne vulgaris [22,23,24]. Using restricted maximum likelihood (REML) with Hartung–Knapp adjustment as the primary estimator, we observed a modest reduction in inflammatory lesion counts favoring probiotics (SMD −0.57; 95% CI −0.94 to −0.21) [18,19]. Sensitivity analyses—including DerSimonian–Laird estimation and leave-one-out procedures—yielded comparable results, suggesting directional stability despite the limited evidence base [34,35]. No serious adverse events were reported across trials, indicating a generally favorable short-term safety profile [22,23,24].

### 4.1. Interpretation in Context of Existing Evidence (PRISMA 23a)

Our findings are broadly consistent with earlier, smaller meta-analyses and extend the literature by incorporating more recent randomized trials (2024–2025) and employing contemporary variance-estimation methods [22,23,24,32,33,34,35]. Mechanistic studies have suggested that selected probiotic strains may influence acne-related pathways—such as reducing pro-inflammatory cytokines (IL-1β, IL-6, TNF-α), enhancing epithelial barrier function, and modulating IGF-1/mTORC1 signaling—although these mechanistic insights are not sufficient to infer clinical benefit on their own [25,26,27,28,30].

The modest pooled clinical effect observed in this analysis may be compatible with these mechanistic observations; however, the 95% prediction interval (−1.25 to 0.11) indicates that true effects in future settings could range from substantial improvement to minimal or no benefit [36]. This wide interval likely reflects genuine clinical and methodological heterogeneity, including differences in probiotic strain composition, single- versus multi-strain formulations, dosing regimens, delivery matrices, baseline acne severity, and the extent of concomitant topical or systemic therapy use [25,29,30].

Given that the synthesis is based on only three heterogeneous trials, these findings should be considered preliminary and hypothesis-generating, rather than definitive evidence for routine probiotic use in acne management. Larger, standardized trials with harmonized outcome measurement will be required to clarify both the magnitude and consistency of any probiotic-associated benefit.

### 4.2. Limitations of Included Evidence (PRISMA 23b)

The certainty of evidence in this review is constrained by several important limitations. First, the evidence base consists of only three double-blind randomized controlled trials, which limits the precision of pooled estimates and reduces the reliability of heterogeneity metrics, publication bias assessments, and any subgroup or sensitivity analyses. Second, substantial heterogeneity (I^2^ = 72%) was present, reflecting genuine clinical and methodological diversity across trials rather than purely statistical variation. The probiotic formulations differed markedly in strain composition (single- vs. multi-strain products), dosing regimens, viability, delivery matrices, and treatment durations, and these differences likely influenced treatment responsiveness. Moreover, trials using multi-strain formulations co-administered with topical or systemic agents may not be directly comparable to single-strain monotherapy interventions.

Variability in baseline acne severity, the timing of outcome assessments, and the specific scoring tools used (despite all reporting inflammatory lesion counts) further complicates synthesis and may contribute to the wide prediction interval, which encompasses the possibility of no clinically meaningful effect. Third, the limited geographic representation—with two studies conducted in Asia and one in Europe—restricts generalizability to other populations, including those with differing genetic backgrounds, dietary patterns, microbiome profiles, and healthcare practices.

Collectively, these factors underscore the need for cautious interpretation of the pooled SMD and support viewing the current evidence as preliminary and hypothesis-generating. Larger, more standardized trials are needed to reduce heterogeneity, improve precision, and strengthen confidence in the clinical implications of probiotic supplementation for acne.

### 4.3. Influence of Study Design Differences on Inconsistency

The included trials differed substantially across several methodological domains, and these variations likely contributed to the inconsistency observed in the pooled results. Population characteristics varied, including differences in age distribution, baseline acne severity, duration of disease, and the relative proportions of comedonal versus inflammatory phenotypes. Such differences can meaningfully alter treatment responsiveness and plausibly account for some of the between-study variability.

Intervention protocols also differed. Trials used distinct probiotic formulations (single- vs. multi-strain combinations), dosing regimens, delivery matrices, and treatment durations ranging from 4 to 12 weeks. The use of concomitant topical therapies or variable skincare instructions further introduced non-uniform exposure conditions that reduce comparability across studies.

In addition, outcome measurement strategies were not standardized. Although inflammatory lesion counts were consistently reported, the timing of assessments and the acne grading instruments used (e.g., lesion counts, global severity scales) varied, and assessor blinding procedures differed. These discrepancies introduce measurement heterogeneity that can inflate between-study inconsistency.

Finally, variability in risk-of-bias domains—including allocation concealment, participant and assessor blinding, and incomplete outcome reporting—may contribute to dispersion in effect sizes. Trials with higher risk of bias have been shown in other contexts to produce larger or more unstable estimates.

Taken together, these design differences likely acted as important effect modifiers and provide a coherent explanation for the substantial heterogeneity observed in this meta-analysis. Future trials employing standardized severity grading, harmonized treatment durations, well-characterized probiotic formulations, and rigorously blinded outcome assessments would improve comparability and strengthen confidence in pooled estimates.

#### 4.3.1. Sensitivity Analysis Explanation

Several sensitivity analyses were performed to evaluate the robustness of the pooled effect. Leave-one-out analysis demonstrated that removal of any single study produced only modest fluctuations in the magnitude of the pooled SMD and did not alter the direction of the effect, indicating that no individual trial disproportionately influenced the results. Similarly, analyses excluding studies with higher risk-of-bias ratings yielded effect estimates consistent with the primary analysis, suggesting that methodological quality alone did not drive the observed variability.

Re-synthesizing the data using alternative estimators—including fixed-effect, DerSimonian–Laird, and Hartung–Knapp–adjusted random-effects models—produced convergent results, further supporting analytic stability. Restricting the analysis to trials employing more standardized outcome assessment scales did not materially change the pooled estimate.

Taken together, these sensitivity analyses reinforce the directional robustness of the findings while acknowledging the inherent limitations imposed by the small evidence base.

#### 4.3.2. Subgroup Analysis Rationale (Severity, Duration, Dosage)

Subgroup analyses were conducted to explore whether differences in clinical or methodological characteristics could plausibly account for the observed heterogeneity. Baseline acne severity was examined because treatment responsiveness is known to vary across mild, moderate, and severe disease, and combining populations with divergent prognoses may obscure differential effects.

Treatment duration (<8 weeks vs. ≥8 weeks) was evaluated as a potential modifier based on established therapeutic timelines for acne interventions, many of which require prolonged administration to achieve maximum benefit. Differences in intervention duration may therefore influence the magnitude and timing of clinical response.

Finally, dosage- and concentration-based comparisons were explored to assess whether higher-strength or multi-strain probiotic formulations exerted systematically different effects. Although these subgroup analyses were exploratory and constrained by the small number of available trials, the selected categories reflect biologically plausible and clinically relevant dimensions of variability grounded in established pharmacologic principles.

### 4.4. Limitations of the Review Process (PRISMA 23c)

While this review adhered closely to PRISMA 2020 guidelines [37] and employed dual independent screening, data extraction, and risk-of-bias assessment [38], several review-level limitations should be acknowledged. First, small-study bias assessments are inherently underpowered when fewer than ten studies are available, limiting confidence in the results of Egger’s test and any visual interpretation of the funnel plot [40,41]. Second, although the search strategy was comprehensive across major bibliographic databases and clinical trial registries, the possibility of unpublished or selectively reported negative trials cannot be excluded and may influence the observed effect estimates.

Third, for one included study, conversion of medians to means was required to enable synthesis. Although validated statistical methods were applied [36], such transformations may introduce minor imprecision. Finally, meta-regression and formal subgroup analyses could not be conducted because only three trials met the inclusion criteria [32,33,34,35,39], limiting the ability to systematically explore effect modifiers.

Overall, these constraints highlight the challenges of evidence synthesis in a developing research area and emphasize the need for larger, prospectively registered, and methodologically standardized randomized trials.

### 4.5. Implications for Clinical Practice, Policy, and Research

Although the findings suggest that oral probiotics may have a potential adjunctive role in acne management, several factors limit their immediate applicability in real-world clinical settings. First, probiotic formulations are not standardized, and the products used in the included trials varied widely in strain composition, colony-forming units, viability, excipients, delivery vehicles, and treatment durations. Many of these formulations are proprietary or not commercially available, making direct translation into routine practice challenging. At present, there is no consensus on an optimal probiotic “regimen” for acne.

Second, regulatory oversight differs substantially across countries. In most jurisdictions, probiotics are regulated as foods or dietary supplements rather than as pharmaceutical agents. As a result, product quality, purity, labeling accuracy, and viable CFU counts can vary considerably between manufacturers. Independent analyses frequently reveal discrepancies between stated and actual microbial content, raising concerns about reproducibility and consistency of therapeutic effects. Mandatory manufacturing standards for strain identity, stabilization, and potency are not universally required, further complicating clinical implementation.

Third, the trial environments typically involved motivated participants under close follow-up, which may not reflect adherence patterns, concomitant skincare behaviors, or real-world co-medication use. Consequently, clinicians should exercise caution when extrapolating these results to over-the-counter products, to patients with different demographic or clinical characteristics, or to settings where product quality cannot be assured.

From a research standpoint, these limitations emphasize the need for strain-specific, adequately powered, and methodologically standardized randomized trials with harmonized outcome measures. Improved regulatory frameworks, quality-control standards, and reporting guidelines would also strengthen the clinical translation of probiotic-based therapies. Policymakers may consider establishing clearer classification and labeling requirements for probiotics intended for therapeutic use to enhance safety, efficacy, and consumer confidence.

### 4.6. Future Research Directions

Future studies should aim to strengthen the evidence base by addressing several important methodological gaps identified in this review. First, probiotic interventions should be standardized, with clearly defined strain identities, verified colony-forming unit (CFU) counts, delivery matrices, and treatment durations. Rigorous strain-level characterization and stability testing are essential, as variability in formulation likely contributes to the heterogeneity observed across current trials.

Second, future RCTs should incorporate mechanistic endpoints to elucidate pathways through which probiotics may influence acne, including gut and skin microbiome sequencing, short-chain fatty acid profiling, assessments of epithelial barrier integrity, and biomarkers related to IGF-1 and mTORC1 signaling. Integrating mechanistic and clinical outcomes will help clarify the biological plausibility of probiotic effects.

Third, harmonized acne severity assessments are needed. Standardized lesion-based counts or validated instruments—such as the Global Acne Grading System (GAGS) or Investigator’s Global Assessment—would substantially improve comparability across trials and enable more accurate quantitative synthesis.

Fourth, adequately powered multicenter randomized controlled trials are required to enhance precision, explore heterogeneity, and allow meaningful subgroup analyses across age groups, acne severity strata, and treatment settings. Trials should also evaluate probiotics as adjunctive therapies, particularly in combination with topical retinoids, benzoyl peroxide, or antibiotic-stewardship regimens, which reflect common real-world treatment paradigms.

Overall, although current evidence suggests that oral probiotics may confer a modest clinical benefit with a favorable short-term safety profile, larger, standardized, and mechanistically informed trials are needed to improve certainty and guide evidence-based clinical recommendations.

## 5. Conclusions

In this PRISMA 2020–compliant systematic review and meta-analysis of three double-blind randomized controlled trials, oral probiotic supplementation was associated with a modest reduction in inflammatory acne severity. Directional consistency across multiple sensitivity analyses supports the stability of this finding. No serious adverse events were reported, indicating a generally favorable short-term safety profile.

However, the evidence base remains small and methodologically heterogeneous, with marked variation in probiotic strains, formulations, dosing regimens, treatment durations, and concomitant therapies. The 95% prediction interval (−1.25 to 0.11) encompassed the possibility of minimal or no effect, suggesting that true clinical responses may vary substantially across populations, products, and care settings. Accordingly, the certainty of evidence is low-to-moderate, and current findings should be regarded as preliminary and hypothesis-generating rather than definitive.

Given rising concerns regarding antimicrobial resistance and antibiotic-associated dysbiosis, probiotics may represent a generally safe, potentially antibiotic-sparing adjunctive option within comprehensive acne management. Nonetheless, translation to routine practice should remain cautious and individualized until stronger evidence becomes available.

To confirm and refine these observations, larger, adequately powered, and standardized multicenter RCTs are needed. Such trials should use harmonized severity measures and incorporate mechanistic endpoints—such as microbiome profiling, inflammatory biomarkers, and IGF-1/mTOR signaling assays—to clarify the pathways through which probiotics may influence acne. Only with more rigorous and consistent evidence can probiotic supplementation be reliably evaluated for routine clinical recommendation.

## Figures and Tables

**Table 1:** Summary of the Cochrane Risk of Bias 2.0 (RoB 2.0) [23].

Study	Randomization	Deviations from Interventions	Missing Data	Outcome Measurement	Reported Results	Overall Risk
Kim 2021 [24]	Low	Low	Low	Low	Low	Low
Eguren 2024 [22]	Low	Low	Low	Low	Low	Low
Atefi 2025 [23]	Low	Some concerns	Low	Some concerns	Low	Some concerns

**Table 2:** GRADE evidence profile summarizing the certainty of evidence for each of the three included randomized controlled trials evaluating oral probiotics for acne vulgaris.

Study	Outcome	Risk of Bias	Inconsistency	Indirectness	Imprecision	Publication Bias	Overall Certainty
Kim 2021 [24]	Change in inflammatory lesion count	No downgrade (Low risk)	Downgraded (I^2^ = 72%)	No downgrade	Downgraded (small N)	No downgrade	MODERATE
Eguren 2024 [22]	Change in inflammatory lesion count	No downgrade (Low risk)	Downgraded (I^2^ = 72%)	No downgrade	Downgraded (small N)	No downgrade	MODERATE
Atefi 2025 [23]	Change in inflammatory lesion count	Downgraded (Some concerns)	Downgraded (I^2^ = 72%)	No downgrade	Downgraded (small N)	No downgrade	LOW

**Table 3:** Key characteristics of the three double-blind randomized controlled trials included in the systematic review [22,23,24].

Study	Year	Region	Sample Size (*n*)	Duration (Weeks)	Probiotic Intervention	Comparator	Primary Outcome	Secondary Outcomes
Kim 2021 [24]	2021	Asia	80	12	*Lactobacillus plantarum CJLP55* (multi-strain)	Placebo	Inflammatory lesion count	Non-inflammatory lesions; Total lesion count; Adverse events
Eguren 2024 [22]	2024	Europe	90	10	*Lactobacillus paracasei* (single strain)	Placebo	Inflammatory lesion count	Global severity scale; Total lesions; Adverse events
Atefi 2025 [23]	2025	Asia	61	8	*Bifidobacterium lactis* + *Lactobacillus acidophilus* (multi-strain)	Adjunct topical therapy	Inflammatory lesion count	Non-inflammatory lesions; Total lesion count; Safety outcomes

## Data Availability

All extracted data, analytic scripts, and Supplementary tables are available within the Supplementary Materials and upon reasonable request from the corresponding author (J.-W.T.).

## Supplementary Materials

The following supporting information can be downloaded at: https://www.mdpi.com/article/10.3390/medicina61122152/s1, Table S1: Full-text Articles Excluded After Eligibility Assessment and Reasons for Exclusion [3,18,26,27,28,29,43,44,45,46,47,48,49,50,51,52,53,54,55,56,57].

## Abbreviation

The following abbreviation is used in this manuscript:

RoB	Risk of Bias

## Appendix A

Appendix A provides the completed PRISMA 2020 Checklist for the full manuscript. Each of the 27 PRISMA items is documented with corresponding locations in the text, confirming adherence to all reporting standards required for systematic reviews and meta-analyses. This checklist details compliance across study rationale, search strategy, selection process, data collection, risk-of-bias assessment, synthesis methods, reporting biases, and certainty of evidence. The completed checklist ensures transparency and reproducibility of all methodological steps undertaken in this review.

**Table A1:** Completed PRISMA 2020 Checklist outlining compliance with all 27 reporting items required for systematic reviews and meta-analyses. Each item is cross-referenced to its location within the manuscript to ensure transparency and adherence to PRISMA reporting standards.

Item #	Checklist Item	Completed?	Location in Manuscript
1	Identify as systematic review	Yes	Title Page/Abstract
2	Structured abstract	Yes	Abstract
3	Rationale	Yes	Introduction
4	Objectives	Yes	Introduction (final paragraph)
5	Eligibility criteria	Yes	Methods 2.2
6	Information sources	Yes	Methods 2.3
7	Search strategy	Yes	Appendix C
8	Selection process	Yes	Methods 2.4
9	Data collection process	Yes	Methods 2.5
10	Outcomes definition	Yes	Methods 2.7
11	Risk of bias methods	Yes	Methods 2.6
12	Effect measures	Yes	Methods 2.7
13	Synthesis methods	Yes	Methods 2.8
14	Reporting bias assessment	Yes	Methods 2.9
15	Certainty assessment	Yes	Methods 2.10
16	Study selection results	Yes	Results 3.1 + Figure 1
17	Study characteristics	Yes	Results 3.2 + Table 3
18	Risk of bias results	Yes	Results 3.3 + Table 1
19	Results of individual studies	Yes	Results 3.4 + Figure 2
20	Results of syntheses	Yes	Results 3.4–3.5
21	Reporting bias results	Yes	Results 3.6 + Figure 3
22	Certainty of evidence	Yes	Results 3.8
23	Discussion of results	Yes	Discussion Section 4
24	Limitations of evidence	Yes	Discussion 4.2
25	Limitations of review processes	Yes	Discussion 4.3
26	Implications for practice	Yes	Discussion 4.4
27	Registration & protocol	Yes	Methods 2.1 (PROSPERO)

## Appendix B

Appendix B provides the completed PRISMA 2020 Abstract Checklist. Each of the 12 PRISMA Abstract items is documented with the corresponding location in the structured abstract, confirming full adherence to required reporting standards for systematic reviews and meta-analyses. The checklist demonstrates compliance across key elements, including identification of the study as a systematic review, objectives, eligibility criteria, information sources, risk of bias assessment, synthesis methods, main results, limitations, and registration information. This completed checklist ensures accuracy, transparency, and completeness of abstract-level reporting.

**Table A2:** Completed PRISMA 2020 Abstract Checklist outlining compliance with all 12 required reporting items for structured abstracts of systematic reviews and meta-analyses, with corresponding locations indicated within the manuscript abstract.

Item #	Checklist Item	Completed?	Where Reported
1	Identification as a systematic review	Yes	Abstract—Opening sentence
2	Objectives	Yes	Abstract—Background and Objectives
3	Eligibility criteria	Yes	Abstract—Methods
4	Information sources and last search date	Yes	Abstract—Methods
5	Risk of bias assessment	Yes	Abstract—Methods
6	Synthesis methods	Yes	Abstract—Methods
7	Included studies and participants	Yes	Abstract—Results
8	Effect of intervention	Yes	Abstract—Results
9	Limitations of evidence	Yes	Abstract—Conclusions
10	Interpretation	Yes	Abstract—Conclusions
11	Funding	Yes	Abstract—Funding statement
12	Registration	Yes	Abstract—Registration line (PROSPERO)

## Appendix C

### Appendix C.1

#### Appendix C.1.1. PubMed Search Strategy (Last Updated: November 2025)

Database: PubMed (MEDLINE) Date of final search: 4 November 2025 Filters applied: None (no language or date limits) (acne [MeSH Terms] OR acne vulgaris [Title/Abstract] OR “acne” [Title/Abstract] OR “acne lesions” [Title/Abstract] OR “inflammatory acne” [Title/Abstract]) AND (probiotics [MeSH Terms] OR probiotic* [Title/Abstract] OR “Lactobacillus” [Title/Abstract] OR “Bifidobacterium” [Title/Abstract] OR “gut microbiota” [Title/Abstract] OR “gut-skin axis” [Title/Abstract]) AND (randomized controlled trial [Publication Type] OR randomized [Title/Abstract] OR randomized [Title/Abstract] OR placebo [Title/Abstract] OR “double blind” [Title/Abstract] OR “double-blind” [Title/Abstract])

#### Appendix C.1.2. Embase (Elsevier) Search Strategy

Database: Embase Date of final search: 4 November 2025 Emtree terms and text words used: (‘acne vulgaris’/exp OR ‘acne vulgaris’ OR acne:ti,ab OR ‘inflammatory acne’:ti,ab) AND (‘probiotic agent’/exp OR probiotic*:ti,ab OR lactobacillus:ti,ab OR bifidobacterium:ti,ab OR ‘gut microbiome’:ti,ab OR ‘gut skin axis’:ti,ab) AND (random*:ti,ab OR ‘double blind’:ti,ab OR placebo:ti,ab OR ‘controlled clinical trial’/exp) No filters applied. Conference abstracts excluded.

#### Appendix C.1.3. Web of Science Core Collection

Database: Web of Science Date of final search: 4 November 2025 Search string: TS = (acne OR “acne vulgaris” OR “inflammatory acne”) AND TS = (probiotic* OR Lactobacillus OR Bifidobacterium OR “gut microbiota” OR “gut-skin axis”) AND TS = (“randomized” OR “double-blind” OR placebo) All years included; no region or language restrictions.

#### Appendix C.1.4. ClinicalTrials.gov Search Strategy

Registry: ClinicalTrials.gov Date of final search: 4 November 2025 Search terms: *Condition*/*Disease*: Acne OR Acne Vulgaris *Other terms*: Probiotic OR Lactobacillus OR Bifidobacterium All trials labeled as *Completed*, *Active*, or *Terminated* were screened.

#### Appendix C.1.5. Additional Search Procedures

The reference lists of all eligible studies and relevant reviews were hand-searched. Forward citation tracking was performed using Google Scholar. No unpublished data from authors were received after inquiry. No automation tools were used to replace manual screening or extraction.

### Appendix C.2. Summary Statement

A comprehensive, reproducible search across four major databases identified 1452 records. All strategies were developed and executed in accordance with PRISMA 2020 and PROSPERO protocol specifications.
