# Supplementary material for: Oral Probiotics in Acne vulgaris: A Systematic Review and Meta-Analysis of Double-Blind Randomized Clinical Trials
Source: Medicina (Kaunas). 2025 Dec 3;61(12):2152. doi: 10.3390/medicina61122152 (PMC12734792; doi:10.3390/medicina61122152)
Supplement: Supplementary file 1 [file medicina-61-02152-s001.zip › medicina-3998505-supplementary.pdf]

Supplementary Table S1.
Full-text Articles Excluded After Eligibility Assessment and Reasons for Exclusion.

No.	Study (Author, Year)	Reason for Exclusion
1	Goodarzi et al., 2020 [18] (*Dermatologic Therapy*) – *Potential of probiotics for treating acne*	Not RCT
2	Mottin et al., 2018 [43] (*Int J Dermatol*) – *Use of probiotics in acne & AD*	Not RCT
3	Sánchez-Pellicer et al., 2022 [3] (*Microorganisms*) – *Acne, microbiome & probiotics*	Not RCT
4	Sutema et al., 2024 [44] (*J Exp Pharmacol*) – *Probiotic supplements & topical applications*	Not RCT
5	De Almeida et al., 2023 [45] (*Microorganisms*) – *Probiotics/postbiotics in skincare*	Not RCT
6	Chilicka et al., 2022 [46] (*Life*) – *Microbiome & probiotics in acne*	Not RCT
7	Shields et al., 2023 [47] (*JAMA Dermatology*) – *Oral nutraceuticals for acne*	Not RCT
8	Boby et al., 2024 [48] (*Arch Dermatol Res*) – *Using probiotics to treat acne*	Not RCT
9	Navarro-López et al., 2021 [49] (*Microorganisms*) – *Probiotics in dermatologists' arsenal*	Not RCT
10	Searle et al., 2025 [50] (*Clin Exp Dermatol*) – *Modulation of microbiome in acne*	Not RCT
11	Mahmud et al., 2022 [51] (*Gut Microbes*) – *Impact of gut microbiome on skin health*	Not RCT
12	Yu et al., 2019 [29] (*Br J Dermatol*) – *Probiotics in dermatology*	Not RCT
13	Sathikulpakdee et al., 2022 [52] *(J Cosmet Dermatol)*-Topical lactobacillui lotion	Topical only
14	Cui et al., 2022 [53] *(Frontiers in Medicine)*-Topical lotion	Topical only
15	Cui et al., 2023 [54] *(Scientific Reports)* – Topical ferment lysate	Topical only
16	Lebeer et al., 2022 [55] *(Cell Reports Medicine)* – Topical lactobacilli	Topical only
17	Cui et al., 2022 [56] (*Indian J Dermatol*) – *Topical anti-acne cream*	Topical only
18	Espinoza-Monje et al., 2021 [57] (Microorganisms) – Weissella viridescens	Topical only
19	Fabbrocini et al., 2016 [26] (Benef Microbes) – *L. rhamnosus SP1*	Incomplete data
20	Gueniche et al., 2014 [27] (*Benef Microbes*) – *L. paracasei NCC2461*	Incomplete data
21	Groeger et al., 2013 [28] (*Gut Microbes*) – *B. infantis 35624 immunologic study*	Incomplete data
